# Effect of Single-Session Aerobic Exercise with Varying Intensities on Lipid Peroxidation and Muscle-Damage Markers in Sedentary Males

**DOI:** 10.5539/gjhs.v4n4p48

**Published:** 2012-05-23

**Authors:** Daruosh Moflehi, Lian-Yee Kok, Tengku-Fadilah T-K, Saidon Amri

**Affiliations:** 1 Department of Sport Studies, Faculty of Education, Universiti Putra Malaysia, Serdang, Selangor, Malaysia; 2 Faculty of Physical Education and Sport Science, Shahid Bahonar University, Kerman, Iran

**Keywords:** single-session aerobic exercise, exercise intensity, malondialdehyde, creatine kinase

## Abstract

**Objectives:**

This study was conducted to evaluate the effect of the different intensity levels of single-session aerobic exercise on serum levels of lipid peroxidation and muscle damage markers in sedentary males.

**Method:**

Fifty one sedentary healthy males aged 21.76±1.89 years were randomly divided into four groups, with one control (n=10) and three treatment groups that attended single-session aerobic exercise with low (n=14), moderate (n=14), and high (n=13) intensities. The serum levels of malondialdehyde (MDA) and creatine kinase (CK) were measured.

**Results:**

Data analysis revealed a significant effect by the intensity levels of aerobic exercise on MDA (*P*=0.001) and CK (*P*=0.003) post-test when the participants in the treatment groups were compared with the control. When the intensity of aerobic exercise was increased, the amount of MDA and CK was also found to be increased.

**Conclusion:**

Single-session aerobic exercise can increase the amount of MDA and CK, suggesting that low intensity level of aerobic exercise should be utilized for more adaptation, and to prevent lipid peroxidation and muscle damage in sedentary males.

## 1. Introduction

Physical activity and regular non-exhaustive exercise have beneficial effects in preventing several chronic diseases such as cardiovascular disease, diabetes, cancer, hypertension, obesity, depression, osteoporosis, and premature death (Powers, Nelson, & Hudson, 2011; Gomez-Cabrera, Domenech, & Viña, 2008). Physical activity includes muscular force along with oxygen consumption (Kirschvink, Moffarts, & Lekeux, 2008). However, as muscular force and/or oxygen consumption increase, reactive oxygen species (ROS), which are oxygen centred free radicals, will be produced in skeletal muscles, liver, and heart during exhausting aerobic exercise (Al Ghouleh, Khoo, Knaus, Griendling, Touyz, Thannickal et al., 2011; Bloomer, Falvo, Fry, Schilling, Smith, & Moore, 2006). This will result in oxidative stress hurting cell tissues in different ways. It has been reported that oxidative stress not only causes direct damage in lipid, protein, and deoxyribonucleic acids (DNA) through free radicals (Alessio, Hagerman, Fulkerson, Ambrose, Rice, & Wiley, 2000), but also works as an inflammation regulator (Kvietys & Granger, 2012; Aoi, Naito, Takanami, Kawai, Sakuma, Ichikawa et al., 2004). It has also been reported that oxidative stress and muscle injury response to equally matched anaerobic exercise is minimal and not different between two modes of training (Bloomer et al., 2006).

Generation of free radicals is influenced by differences in energy requirement, oxygen consumption, and mechanical loads on soft tissue during exercise. Moreover, after unaccustomed exercise, exercise-induced muscle damage is common especially if the exercise is exhaustive or includes eccentric muscle actions (Park, 2006; Sorichter, Puschendorf, & Mair, 1999). In addition, the formation of free radicals results in lipid peroxidation during aerobic exercise which may cause cell and muscle damage. Malondialdehyde (MDA) has mostly been used as an end-product marker of lipid peroxidation (Bailey, Mceneny, Mathieu-Costello, Henry, James, Mccord et al., 2010). MDA levels during exercise are correlated with creatine kinase (CK) which is an indicator of muscle damage (Guzel, Hazar, & Erbas, 2007). Several studies have indicated that following exercise, levels of lipid peroxidation and muscle-damage markers such as MDA and CK increase (Skenderi, Tsironi, Lazaropoulou, Anastasiou, Matalas, Kanavaki et al., 2008; Guzel et al., 2007), but these increases seemed to be evoked only when participants performed at maximal intensity and not at lower intensities (Rahnama, Gaeini, & Hamedinia, 2007; Bloomer, Goldfarb, Wideman, Mckenzie, & Consitt, 2005).

Most of the previously mentioned studies (Lamprecht, Hofmann, Greilberger, & Schwaberger, 2009; Mergener, Martins, Antunes, Da Silva, Lazzaretti, Fontanive et al., 2009; Radovanovic, Bratic, Nurkic, Cvetkovic, Ignjatovic, & Aleksandrovic, 2009; Rahnama et al., 2007; Nosaka & Newton, 2002; Nosaka & Clarkson, 1994) have examined participates through multiple training sessions. Few researches have focused on single-session activities and their effects on the level of lipid peroxidation and muscle damage markers (Mendham, Donges, Liberts, & Duffield, 2011; Guzel et al., 2007). In single session exercise, the participants had not enough time for adaptation to a variety of factors such as mechanical stresses which may increase mitochondrial activity resulting in the production of MDA and the leakage of CK in blood circulation and finally muscle damage (Baechle & Earle, 2008; Feasson et al., 2002; Jackson, 1999). Therefore, the present study was conducted to investigate the effects of single-session aerobic exercise with different intensities, low, moderate, and high on the rate of changes in MDA and CK markers in sedentary males.

## 2. Methodology

### 2.1 Subjects

From a population of 600 students of the Shahid Bahonar University of Kerman in Iran, 51 sedentary male-students (21.76±1.89 yr) who were healthy, had no history of regular exercise for at least 6 months, did not consume any supplements such as vitamin A, C, and E before and during the exercise session, were randomly selected. They were also examined by the state Sport Medicine Center for any cardiovascular or muscle injuries. All participants were asked to avoid performing any strenuous physical activity three days prior to the exercise session. They were informed about the objectives of the study and gave informed consent before starting the experiment. Ethics approved was also obtained from the university.

### 2.2 Aerobic Training

The subjects were randomly divided into a control group (n=10) and three single-session aerobic training groups tested under low intensity (n=14), moderate intensity (n=14) and high intensity (n=13) conditions during aerobic exercise. After warming up for 10 minutes on a cycle ergometer and performing dynamic stretching activities, the participants in the training groups were subjected to 20 min of aerobic treadmill running at low (40% VO_2_ max), moderate (60% VO_2_ max), and/or high (80% VO_2_ max) intensities. Functional capacity (VO_2_ max) was estimated continuously via calculations of the heart-rate reserve (HRR) using a polar belt (Polar Electro Oy Profiessorintie 5 FIN-90440 KEMPELE, Finland) to prescribe aerobic exercise intensity (American College of Sport Medicine ACSM). The target heart rates reserves (THRR) of the participants were estimated using the Karvonen formulas (Baechle & Earle, 2008). The participants were asked to go on the treadmill (h/p/cosmos 8 DE 83365, Germany) with zero slope angle and begin walking by pressing the start button. Based on the Balke modified protocol (1959), after each minute, the speed (km/h) was increased until the treadmill speed reached the THRR based on the required intensity.

### 2.3 Blood Sampling and Analysis

Blood samples (5 ml) were taken from an antecubital forearm vein pre-test while participants were fasting and post-test after performing aerobic training on the treadmill. Sera were collected by centrifuging the blood samples at 1500×g for 10 min, transferred into clean tubes and stored at -80°C for further analysis. The serum level of MDA was measured through a colorimetric method, where MDA in reaction with thiobarbituric acid produced a colourful complex which can be measured at the absorbance value of 532 nm. The serum concentration of MDA was calculated using a standard curve which was prepared using a two-fold serial dilution of 1 ml tetramethoxypropane ranging from 2.5 to 80 nmol/ml. Serum level of creatine kinase (CK) was measured using an assay kit (Parsazmon, Iran) according to the manufacturer’s instruction. Briefly, buffer A and B were mixed with a 1:1 ratio to prepare a volume of 500µl. Twenty microliters of the serum samples was added to the mixed buffers, and the absorbance value was measured at 340 nm using an analyzer (Technicon RA-1000-USA) set at 37°C. The mixture of buffer A and B was used blank for the assay.

### 2.4 Statistical Analysis

Dependent variables were analyzed using a multivariate analysis of covariance (MANCOVA). In this model the pre-test was controlled as a covariate. The Bonferroni comparisons multiple test was used to evaluate significant levels of the effects. Statistical significance for MANCOVA was set at p ≤ 0.05.

## 3. Results

As shown in Table 1, the intensity levels of aerobic exercise significantly affected the post-test MDA level [F (3, 45) = 27.210, *P* = 0.001]. Likewise, MANCOVA analysis indicated that the intensity levels of aerobic exercise has a significant effect on post-test CK [F (3, 45) = 5.455, *P* = 0.003]. In order to determine the relationship between the concentrations of MDA and CK markers pre- and post-test, MDA and CK pre-tests scores were considered as covariate factors. The results indicated a high correlation between the MDA pre- and post-test [F (1, 45) = 10.931, *P* = 0.002], whereas no significant correlation was observed between pre-test MDA and post-test CK [F (1, 45) = 0.006, *P* = 0.936], indicating that the observed difference between MDA and CK post-test were due to the effect of the intensity levels of the exercise.

**Table 1:** MANCOVA on the effects of intensity levels of aerobic exercise on MDA and CK post-test score

^1^DV	Intensity level			MDA pre-test			CK pre-test		

	df	F	Sig.	df	F	Sig	df	F	Sig.
MDA post-test	3	27.21	0.001*	1	10.93	0.002*	1	1.53	0.222
CK post-test	3	5.46	0.003*	1	0.01	0.936	1	6.87	0.012*

* Significant at *P*< 0.05;

1 Dependent variable

**Figure 1:**
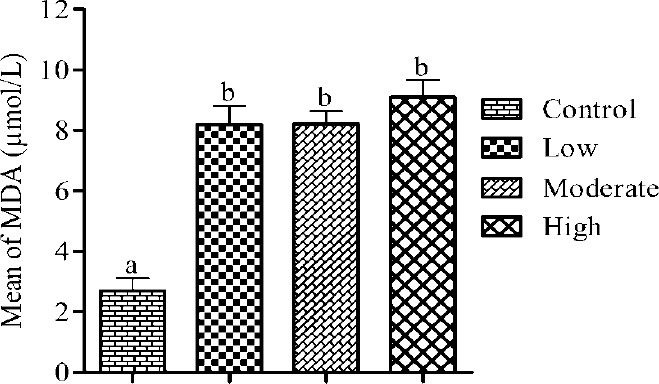
The MDA concentration immediately after aerobic exercise with intensity levels

**Figure 2:**
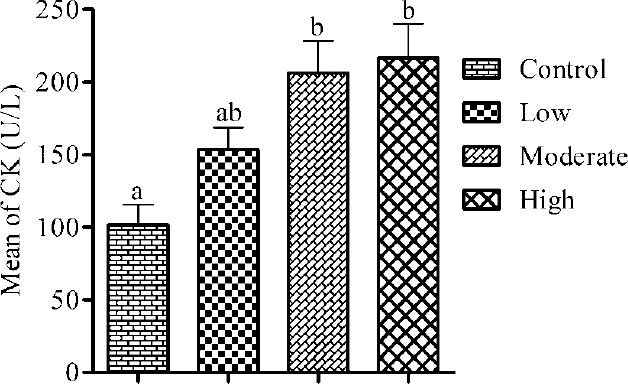
The CK concentration immediately after aerobic exercise with intensity levels

The effect of the intensity levels of aerobic exercise on MDA post-test was assessed using the Bonferroni comparisons multiple tests at a significance level of P ≤ 0.05. The results revealed significant differences among the control and each of the three intensity levels, however, no significant differences among the three intensity levels of aerobic exercise were observed (Table 2) and (Figure 1). The highest amount of MDA post-test was detected in the high intensity level (M = 9.09, SD = 2.08 µmol/L) and the lowest in the control (M = 2.69, SD = 1.32 µmol/L). Data analysis of the results obtained for CK also revealed a significant difference among the controls and the participants in the moderate and high intensity levels of exercise, whereas, no significant difference among the control group and the low intensity level was detected. No significant difference among the intensity levels was also found (Table 2) and (Figure 2). The highest amount of CK post-test was measured in the high intensity level (M = 216.38, SD = 85.62 U/L) and the lowest in the control group (M = 101.60, SD = 43.71 U/L).

**Table 2:** Evaluation MDA and CK among intensity levels of aerobic exercise using Bonferroni test

DV^1^	(I)Intensity Levels		(J)Intensity levels	Mean^2^	S.E.^3^	Sig.
MDA post-test	Control	vs.	Low	-5.380	0.706	0.001*
	Control	vs.	Moderate	-4.926	0.717	0.001*
	Control	vs.	High	-5.928	0.723	0.001*
	Low	vs.	Moderate	0.453	0.652	1.000
	Low	vs.	High	-0.548	0.659	1.000
	Moderate	vs.	High	-1.002	0.652	0.789
CK post-test	Control	vs.	Low	-42.606	28.072	0.816
	Control	vs.	Moderate	-91.723	28.539	0.015*
	Control	vs.	High	-103.559	28.746	0.005*
	Low	vs.	Moderate	-49.117	25.949	0.389
	Low	vs.	High	-60.953	26.210	0.148
	Moderate	vs.	High	-11.836	25.938	1.000

* Significant at *P*< 0.05;

1 Dependent variable;

2 Different means;

3 Standard Error

## 4. Discussion

Unaccustomed and exhaustive aerobic exercise are thought to cause muscle damage by increasing reaction oxygen species (ROS) production in skeletal muscle, liver, and heart and also play major roles in cardiovascular dysfunction (Park, 2006; Taniyama et al., 2003; Sorichter, Puschendorf, & Mair, 1999). Production of free radicals during exercises has an important role in lipid peroxidation and muscle damage. There are several possible mechanisms for the production of free radicals during exercise; hyperoxic injury which may occur in highly intense aerobic exercise and high oxygen uptake and O_2_ flux in an active muscle are important factors in increasing lipid peroxidation and muscle damage, even in highly-trained athletes (Kirschvink et al., 2008; Witt, Reznick, Viguie, Starke-Reed, & Packer, 1992). High oxygen uptake was shown to increase oxygen flux through the mitochondrial electron transport chain, resulting in a decrease in mitochondrial respiratory control, failure of structural integrity of sarcoplasmic reticulum leading to increased lipid peroxidation and release of myoglobin (Mb) and muscle enzymes into the circulation (Kirschvink et al., 2008; Cannon & Blumberg, 2000). Peroxidation of lipids, especially polyunsaturated fatty acids in the cell membrane causes a loss in fluidity as well as increases the permeability which can result in loss of cytosolic proteins and enzymes, and a loss of structural sarcoplasmic reticulum. Both of the above conditions may lead to oxidative damage which has been suggested to be indicative of an increase in the MDA and CK markers (Baker, Bailey, Hullin, Young, & Davies, 2004; Cannon & Blumberg, 2000; Maughan, Donnelly, Gleeson, Whiting, Walker, & Clough, 1989). Elevation of the serum level of CK may be due to the disruption of the sarcometric Z-disk, resulting in the leakage of this protein out of the cell and into the circulation (Feasson, Stockholm, Freyssenet, Richard, Duguez, Beckmann et al., 2002). Damage to lipid membranes can be caused by oxygen-free radicals produced under certain exercise programs (Sen, Packer, & Hiinnincn, 2000), even in aerobic exercise of sufficient intensity (Lovlin, Cottle, Pyke, Kavanagh, & Belcastro, 1987).

The results of this study suggest that lipid peroxidation and muscle damage markers can be affected by the intensity levels of aerobic exercise, where, after a single session of aerobic exercise, significant increases in both MDA and CK was observed. This effect may due to the fact that the participants were sedentary (non-athletes) with no history of regular aerobic exercise, and also did not consume supplements such as antioxidant vitamins (A, C, E) before and during the exercise. Antioxidant supplements can improve antioxidant defences supplied by superoxide dismutase (SOD) against pro-oxidant events (Li, Feng, & Wang, 2005). Furthermore, the participants did not have enough time for adaptation to a variety of factors including mechanical stresses which may increase mitochondrial activity resulting in the production of MDA and the leakage of CK into the blood circulation, and finally muscle damage (Baechle & Earle, 2008; Feasson et al., 2002; Jackson, 1999).

These observations are consistent with previously reported results (Lamprecht et al., 2009; Radovanovic et al., 2009; Seifi-Skishahr, Siahkohian, & Nakhostin-Roohi, 2008; Close, Ashton, Cable, Doran, & Maclaren, 2004) significant increases in MDA and CK levels after exercise programs. For instance, it was reported that there were significant increases in erythrocyte MDA values after 12 weeks of concurrent training (Radovanovic et al., 2009). Another study (Seifi-Skishahr et al., 2008) showed significant increases in MDA concentrations 2 h after high-intensity exercise compared with moderate exercise, and higher post-test CK levels (p<0.05) immediately and 2 h after exercise in both groups. Significant increases in MDA and CK levels have also been reported following downhill running (Close et al., 2004).

However, there are conflicting reports (Mergener et al., 2009; Rahnama et al., 2007; Close, Ashton, Cable, Doran, Holloway, Mcardle et al., 2006; Bloomer et al., 2005) that detected no significant increases in MDA levels after exercise programs. For example, it was reported that there were no significant increases in MDA levels in groups performing muscle building or walking exercise for 45 min at least twice a week (Mergener et al., 2009). Another study (Rahnama et al., 2007) utilised aerobic training that included running at 75% - 80% of maximal heart rate for 20-45 min per day for eight weeks and found no significant changes in MDA levels at rest and after exercise to exhaustion, although an increase in CK was observed in the experimental group. Bloomer et al. (2005) also could not detect a statistical increase in total plasma MDA at any time point post-exercise following either cycling or squatting. Supporting this further was a study (Nosaka & Newton, 2002) that reported no significant increase in the CK level following 8 weeks of eccentric training of the elbow flexors at 80% maximal isometric strength. The divergent results obtained in the different studies may be due to a number of factors such as differences in the duration, intensity, modes of exercise, training status of the participants, age, sex, and even differences in the methods employed for MDA and CK measurement.

## 5. Conclusion

In conclusion, the present study revealed that the intensity levels of aerobic exercise can significantly increase the serum levels of MDA and CK following single-session exercise. The results suggest that by increasing the intensity of exercise, both MDA and CK post-test also increased, indicating that in sedentary people, a single session of high intensity aerobic exercise may increase oxidative stress and lipid peroxidation in skeletal muscle. Therefore, low intensity aerobic exercises can be recommended for sedentary people who want to improve physical fitness without any muscle damage.
